# Generation of uniform-sized multicellular tumor spheroids using hydrogel microwells for advanced drug screening

**DOI:** 10.1038/s41598-018-35216-7

**Published:** 2018-11-21

**Authors:** Jong Min Lee, Da Yeon Park, Letao Yang, Eun-Joong Kim, Christian D. Ahrberg, Ki-Bum Lee, Bong Geun Chung

**Affiliations:** 1 Department of Mechanical Engineering, Sogang University, Seoul, Korea; 2 Department of Biomedical Engineering, Sogang University, Seoul, Korea; 3 Department of Chemistry and Chemical Biology, Rutgers, The State University of New Jersey, New Jersey, USA; 4 Research Center, Sogang University, Seoul, Korea; 5 Department of Life and Nanopharmaceutical Sciences, Graduate School, Kyung Hee University, Seoul, Korea

## Abstract

Even though *in vitro* co-culture tumor spheroid model plays an important role in screening drug candidates, its wide applications are currently limited due to the lack of reliable and high throughput methods for generating well-defined and 3D complex co-culture structures. Herein, we report the development of a hydrogel microwell array to generate uniform-sized multicellular tumor spheroids. Our developed multicellular tumor spheroids are structurally well-defined, robust and can be easily transferred into the widely used 2D culture substrates while maintaining our designed multicellular 3D-sphere structures. Moreover, to develop effective anti-cancer therapeutics we integrated our recently developed gold-graphene hybrid nanomaterial (Au@GO)-based photothermal cancer therapy into a series of multicellular tumor spheroid co-culture system. The multicellular tumor spheroids were harvested onto a two-dimensional (2D) substrate, under preservation of their three-dimensional (3D) structure, to evaluate the photothermal therapy effectiveness of graphene oxide (GO)-wrapped gold nanoparticles (Au@GO). From the model of co-culture spheroids of HeLa/Ovarian cancer and HeLa/human umbilical vein endothelial cell (HUVEC), we observed that Au@GO nanoparticles displayed selectivity towards the fast-dividing HeLa cells, which could not be observed to this extent in 2D cultures. Overall, our developed uniform-sized 3D multicellular tumor spheroid could be a powerful tool for anticancer drug screening applications.

## Introduction

The American Cancer Society predicts 1.6 million new cancer cases and 600,000 Americans to die of cancer in 2017. This corresponds to 1,650 death per day, making cancer the second most common cause of death in the USA^1^. The mortality rates are still high despite a high level of spending on cancer therapy^2^. Despite many new drugs developed with higher anti-cancer therapeutic potential, the key challenge lies in high-throughput screening (HTS) and explicitly understanding the effects of these new drugs in more clinically relevant models. Currently, two-thirds of all drugs that enter Phase II clinical screening and a third of drugs entering Phase III clinical trials fail to transit into the next stage^3^. To obtain a higher success rate in clinical trials, there is an urgent need for the development of drug screening methods which could predict the toxicity and efficacy with higher accuracy and better represent the tumor microenvironment^4^. One promising approach for drug screening applications is the use of three-dimensional (3D) cell culture systems. For example, in conventional two-dimensional (2D) cell culture system, cell-cell and cell-matrix interactions are less prevalent compared to *in vivo* 3D environments, thereby incapable of mimicking tumor microenvironment precisely. Additionally, the cells in 2D cultures can be stretched, which may result in undesired cytoskeletal rearrangements and artificial polarity^5^. In 3D cultures, in contrast, the cell environment can be reproduced with higher accuracy including cell-cell, cell-matrix interactions^6^. *Ma et al*. compared the chemotherapy and nanoparticle penetration properties of different culture systems^7^. They observed that 3D spheroids of HeLa cells displayed similar morphologic characteristics as human solid tumors. 3D spheroids also showed some characteristics of solid tumors, such as resistance to chemotherapeutics that could not be observed in 2D cultures. For spheroids systems to become an alternative to 2D cultures in screening applications, it must be possible to generate 3D spheroids with a reproducible and homogenous size to obtain comparable and reproducible results^8–10^.

Spheroids are typically formed using pellet culture^11^, liquid overlay^12^, hanging drop^13^, spinning flask^14^, and magnetic levitation methods^15^. However, these conventional methods have limitations, such as a lack of reproducibility and a wide distribution of spheroid sizes. To this end, advances in microfabrication techniques provide a promising solution to address these limitations^16^. Microwell arrays can provide a facile method to produce uniform-sized spheroids in a high-throughput manner^17^. For example, concave poly(dimethylsiloxane) (PDMS)-based microwells have been used to generate cancer spheroids which were used for screening anticancer drug-loaded nanoparticles^18^. Due to their biocompatibility, tunable physical and biodegradable properties, hydrogels have widely been used for microwell fabrication^19^. One such example of a synthetic hydrogel are poly(ethylene glycol)(PEG)-based hydrogel microwells, which enable the efficient generation of as well as the culture of uniform-sized embryoid bodies^20^. Alternatively, cell spheroids have also been formed by encapsulating cells into a PEG hydrogel with restricted volumes, which can further achieve a higher level of control over cell-cell interactions^21^. *Lee et al*. found that the spheroid size and functionality could be modulated by the stiffness of the encapsulating hydrogel^22^. Utilizing cell spheroids encapsulating hydrogel particles for self-assembling, macroscopic 3D structures have been further achieved^23^. Still, given that multiple cell types are commonly involved in tissue and tumor genesis, and considering the complex and heterogeneous microenvironment during tumor genesis, it is challenging to utilize previously developed single-cell spheroids for accurately mimicking organs or tumors for precision drug screening. To this end, reliable methods for creating multicellular spheroids are of particular value. For example, HepG2 and NIH3T3 fibroblast cells in spheroids were co-cultured in a digital microfluidic device to better mimic *in vivo* liver functions^24^. Also, progenitor cells and mesenchymal stem cells were co-cultured in PDMS microwells to form spheroids^25^. Moreover, spheroids of rat hepatocytes and fibroblasts were formed on electrospun scaffolds^26^, and colonic adenocarcinoma cells in co-culture with normal colonic fibroblasts were formed using a rotary orbit shaker^27^. Despite the development of these co-culture spheroid systems, the methods are still either labor intensive or produce spheroids in a non-homogeneous manner, which can lead to inconclusive results when applied for screening anti-cancer therapeutics. Therefore, there is an urgent need to develop a high throughput and reliable approach that can produce homogeneous multicellular spheroids.

Recently, nanomaterials (e.g., graphene nanosheet^28^, gold nanoparticle) have been used to explore the cancer biology^29^. Compared to a conventional small molecule-based anti-cancer therapeutics, these nanomaterials with unique physical, chemical and biological properties can not only be integrated into excellent drug delivery platforms, but also can be developed into unique anti-cancer and imaging reagents. Through a further hybridization of two nanomaterials having orthogonal functions using advanced nanotechnology, unique physiochemical properties from each nanomaterial can be integrated into a single nanoparticle platform for advanced biological applications^30^. For example, gold nanoparticles encapsulated by graphene oxide (GO) nanosheets can be used to detect changes on a cellular level using Surface Enhanced Raman Spectroscopy (SERS)^31^, or a gold nanocluster-functionalized reduced GO nanosheets were used for combined drug delivery and imaging of cancer cells^32^. However, despite being a highly potent cancer theragnostic reagent, its therapeutic effects have not yet been evaluated in well-defined 3D culture systems. This is further compounded by the dramatically increased sizes and more heterogeneous surface chemistries from nanomedicinal reagents, which could lead to a higher complexity during drug screening as compared to conventional small molecules. Addressing the aforementioned challenges, we developed a high-throughput hydrogel microwell-based method to generate uniform-sized multicellular tumor spheroids. The multicellular tumor spheroids could be used as a 3D model in high-throughput screening tests, effectively improving the accuracy and accelerating the screening process significantly. We successfully generate two different co-culture systems using the developed microwell array, including HeLa/human umbilical vein endothelial cells (HUVEC) and HeLa/Ovarian cancer cells. While the HeLa/HUVEC co-culture acts as a tumor/endothelium model, HeLa/Ovarian co-culture can be used to simulate a secondary tumor resulting from metastasis as previously observed with cervical cancer patients^33,34^. To demonstrate an application of the spheroids as a model system for tumor screening applications, we further investigate the potential of a novel hybrid gold nanoparticle-functionalized graphene oxide (Au@GO) for photothermal therapy (PTT)-based anti-cancer applications.

## Materials and Methods

### Hydrogel Microwell Fabrication

Hydrogel microwell arrays were fabricated using a PDMS stamp as previously described^20,35^. Briefly, photomasks were designed using Autocad (Autodesk, USA), printed on a photomask, and transferred onto silicon wafers (Wanxiang SiliconPeak Electronics Co., China) using SU-8 negative photoresist (MicroChem Corp., USA) according to manufacturer’s instructions. After ultraviolet (UV) light exposure, wafers were treated with SU-8 developer (MicroChem Corp., USA). PDMS prepolymer solution (10:1, monomer: curing agent, Sylgard 184, Dow Corning Corp., USA) was poured onto the silicon molds, and air bubbles were removed in a vacuum chamber for 30 minutes, followed by curing in an oven at 85 °C for 2 hours. Glass slide, acting as a substrate for supporting the hydrogel microwell array, was then treated with 3-(trimethoxysilyl) propylmethacrylate (TMSPMA, Sigma-Aldrich Co., USA) for 30 minutes and further heated for 1 hour at 70 °C afterwards to provide better adhesion to the hydrogel. An aqueous solution containing 10% (w/w) PEG 1,000 dimethacrylate (Polysciences, USA) and 1% (w/w) of the photoinitiator, 2-hydroxy-2-methyl propiophenone (Sigma-Aldrich Co., USA), were poured between the glass slide and the PDMS stamp. The hydrogel was polymerized by a radical chain growth reaction for 30 seconds using a UV light source (320–350 nm, Omnicures Series 1500, EXFO, Canada). Lastly, the PDMS mold was carefully peeled from the glass slide and hydrogel microwell arrays washed with ethanol followed by overnight storage in phosphate buffered saline (PBS, Thermo Fisher Scientific, USA). A movie in the production process and further use of the microwells can be found in the Supplemental Movie.

### Spheroid Culture

For spheroid culture, green fluorescent protein (GFP)-labeled HeLa cells and red fluorescent protein (RFP)-labeled ovarian cells, were cultured in Dulbecco’s Modified Eagle’s Medium (DMEM, Thermo Fisher Scientific, USA) with 10% fetal bovine serum (FBS, Thermo Fisher Scientific, USA) and 1% penicillin-streptomycin (Thermo Fisher Scientific, USA). Further HUVEC-labeled with cell trace violet cell proliferation kit (Molecular Probes, USA) were cultured in Endothelial Cell Growth Medium-2 (EGM-2, Lonza, Switzerland). The cells were seeded into the hydrogel microwell arrays with a concentration of 1 × 10^6^ cells/mL and incubated for one day at 37 °C. The tumor spheroids formed in the hydrogel microwells were replated onto polystyrene confocal dishes (Ibidi, Germany) and were subsequently incubated for one more day at 37 °C.

### Synthesis of Au@GO Nanoparticles

Au@GO nanoparticles (40 nm diameter) and spherical shape were synthesized from positively charged nanoparticles and negatively charged GO using an electrostatic enabled assembly method^31^. All the reagents used were from Sigma Aldrich. Briefly, to synthesize positively charged nanoparticles, 5.0 mg of the HAuCl_4_ solution was prepared at 4 °C, then 1.0 mL of 3.0 mg/mL cysteamine was quickly added into the gold yellow solution^36^. The solution should turn orange immediately, and then was further stirred at room temperature. After 15 minutes’ stirring, under dark conditions, 15 μL of 3.8 mg/mL NaBH_4_ was quickly injected into the solution under rapid stirring. 1 hour later, the stirring was slowed down, and the reaction was continued overnight to obtain dark red colored cysteamine-functionalized gold (Au-CA) nanoparticles. To synthesize the negatively charged GO, a modified two-step Hummer’s method was used^37^. By adding 1.0 g of graphite, 2.5 g K_2_S_2_O_8_, 2.5 g P_2_O_5_, and 12 mL concentrated H_2_SO_4_ (98%) step by step with caution, the viscous solution was stirred overnight at 80 °C. Then, the mixture was slowly added to 500 mL of distilled water and further stirred overnight. Pre-oxidized graphite was obtained by filtering the 500 mL solution and was dried for 24 hours. To obtain graphite oxide, the pre-oxidized graphite oxide was added into 120 mL H_2_SO_4_ (98%), and 15 g of KMnO_4_ powder was slowly added to the solution under 10 °C, and then the mixture was stirred at 35 °C for 4 hours, followed by a slow addition of 250 mL distilled water. After further stirring for 3 hours, the reaction was quenched by the drop-by-drop addition of 20 mL 30% H_2_O_2_, with the appearance of shining yellow colored graphite oxide. GO was obtained by first purifying graphite oxide by 10% HCl and distilled water, then the graphite oxide was exfoliated by a Brandson ultrasonicator. To synthesize the GO core-shell nanoparticle, 1.0 mL of diluted solution (100 μg/mL) of cysteamine functionalized gold nanoparticle was slowly added (2 mL/h) into 10 mL of concentrated (1.0 mg/mL) of GO under vigorous stirring. After 2 hours, the particles were purified by centrifugation at 10,000 rpm for 10 minutes, followed by distilled water washing for 3 times. The final concentration of nanoparticles was adjusted accordingly by adding the proper amount of water. To characterize the nanoparticles, a Philips CM12 electron microscope with an AMT-XR11 digital camera was used for transmission electron microscopy (TEM) characterization; a Malvern Nano series Zeta sizer was used for measuring the hydrodynamic sizes and surface charges; Agilent Cary 60 UV-Vis was used to measure the absorption spectrum.

### Doxorubicin loading in Au@GO nanoparticles

For loading of Au@GO nanoparticles with doxorubicin 1.0 mg of doxorubicin was dissolved in 1 mL of ultrapure water (Millipore, US) under constant stirring. To this a solution of Au@GO nanoparticles was added drop by drop to the doxorubicin solution under continuous stirring, until the nanoparticle concentration reached 1.0 mg/mL. After 8 hours, 5.0 mL of PBS was injected into the mixture to increase the ionic strength and enhance the loading of doxorubicin. The loaded particles were purified by repeated washing followed by centrifugation at 8,000 rpm for 10 minutes, until the supernatant had no observable color from the doxorubicin. Finally the particles were collected by centrifugation and the pellet re-dispersed in 1.0 mL of medium (10% FBS) which was stored at 4 °C until further use.

### Analysis of doxorubicin-loaded Au@GO nanoparticle diffusion

Diffusion of Au@GO nanoparticles was analyzed using previously generated HeLa/HUVEC spheroids. HeLa/HUVEC spheroids were cultured in co-culture medium (1:1 ratio of 10% FBS in DMEM and HUVEC media) for one day in a 24-well plate. Afterwards the medium was exchanged with medium containing 10 μg/mL Au@GO nanoparticles loaded with doxorubicin and briefly stirred. Fluorescent images of the spheroids were taken after various time points (0, 1, 3, and 5 hours) using a fluorescent microscope (Nikon, Ti series, Japan) and utilizing the auto-fluorescence of the doxorubicin. Before imaging, the media was exchanged with PBS to remove doxorubicin in solution, to reduce the background noise. Fluorescent images were analyzed using imageJ.

### Cell Viability Analysis

The cell viability of 2D cultures was measured using 3-(4,5-Dimethylthiazol-2-yl)-2,5-Diphenyltetrazolium Bromide (MTT) assays (Roche, Germany), cell viabilities were normalized to controls without the addition of nanoparticles. Fluorescent-activated cell sorting (FACS, FACS Calibur, BD Bioscience, USA) analysis was further conducted. FACS results were visualized using the Flow-Jo software (BD Bioscience, USA).

### Photothermal Therapy

After replating the spheroids onto the polystyrene confocal dish and further incubation for one day, the culture medium was exchanged with medium containing 1 vol% Au@GO and was subsequently incubated for one day. Lastly, the spheroids were irradiated with a near-infrared (NIR) laser (808 nm, 5 W/cm^2^, BWF2, B&W, Denmark) for 10 minutes.

## Results and Discussion

### Hydrogel Microwell Fabrication for Multicellular Tumor Spheroids

**Figure 1:**
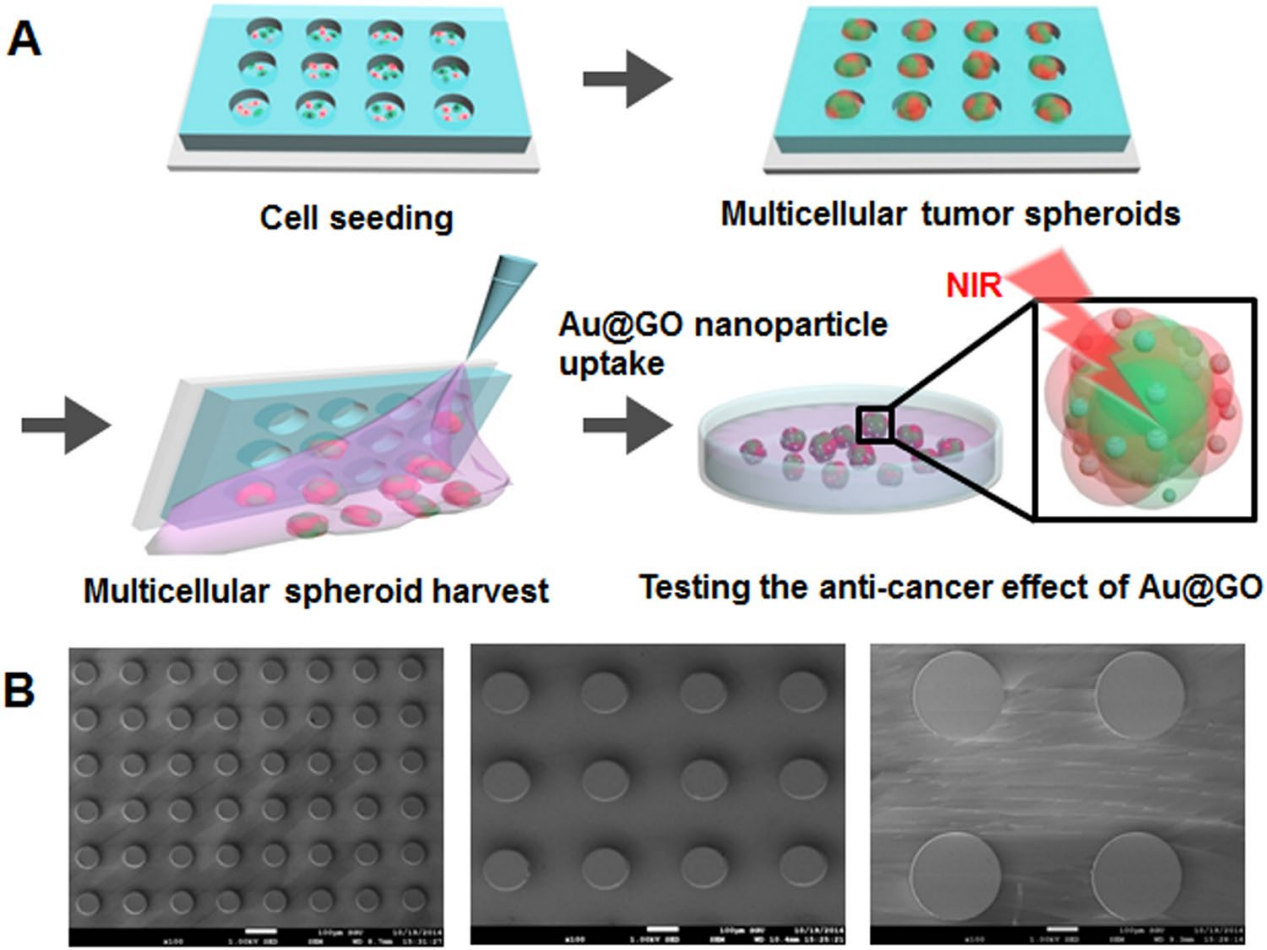
Schematics of uniform-sized 3D tumor spheroid culture inside of PEG hydrogel microwells, spheroid harverst and use of spheroids in for PTT experiments (**A**). SEM images of PDMS stamp for the PEG hydrogels with microwell diameters of 75 (left), 150 (middle), and 300 µm (right) (**B**).

**Figure 2:**
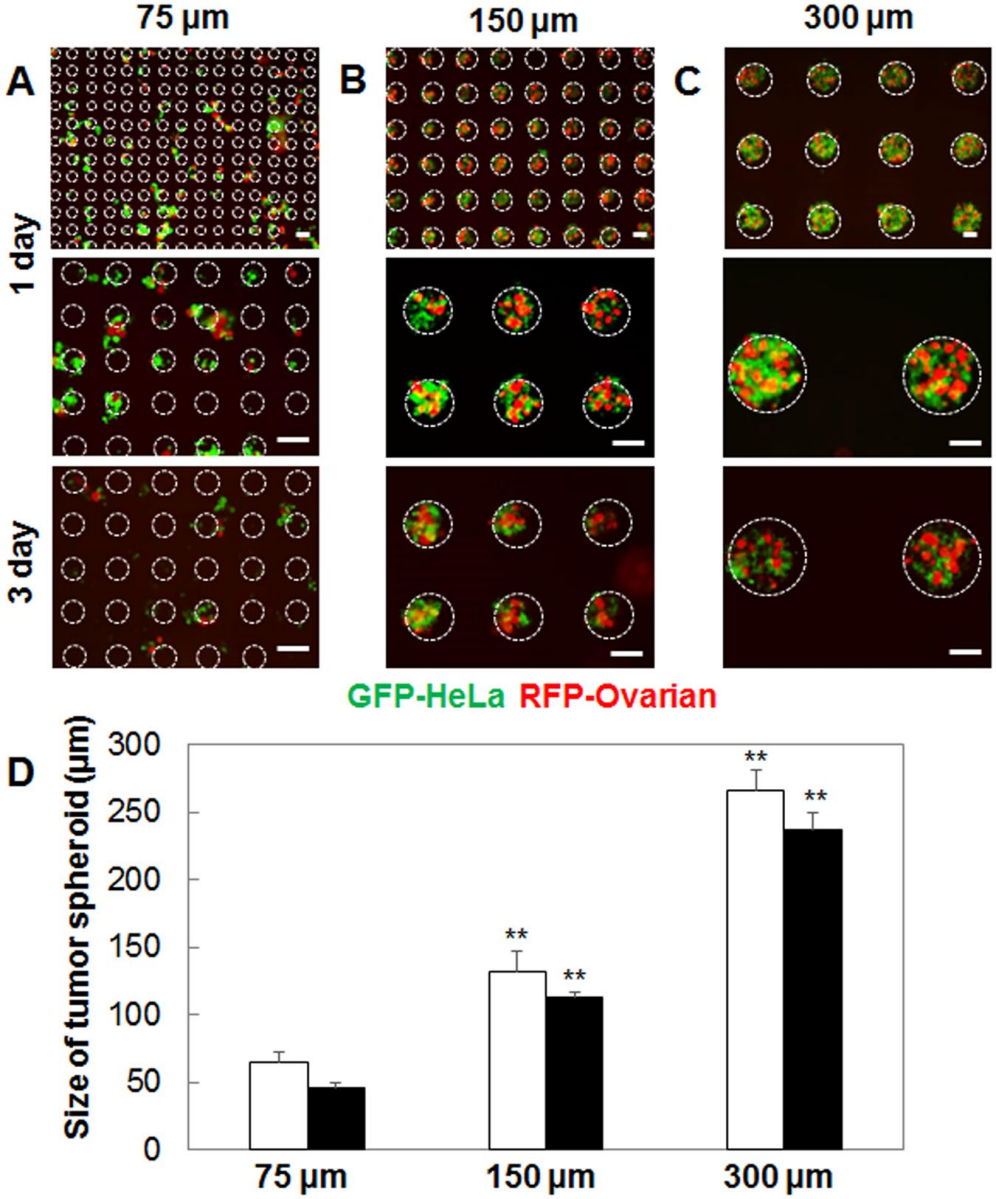
Fluorescent microscopy images of HeLa (green) and Ovarian (red) spheroid co-cultures inside of 75 (**A**), 150 (**B**), and 300 µm microwells (**C**). Scale bars are 100 µm and wells positions are indicated through white circles. Analysis of spheroid diameter after one (white bars) and three days (black bars) of culture inside hydogel microwells with different sizes (**D**).

We developed PEG hydrogel microwells to generate uniform-sized multicellular tumor spheroids (Fig. 1). The PDMS stamps were able to reproduce microwells with diameters of 75, 150, and 300 µm as shown by scanning electron microscopy (SEM) (Fig. 1B). Co-culture of tumor spheroids inside of the hydrogel microwells was facile, and it could be observed that after one day of culture spheroids had formed for all three microwell sizes (75, 150, and 300 µm, Fig. 2A–C). While the cells grew exclusively inside the larger microwells (150 and 300 µm in diameter), cell growth could also be observed around the smallest microwells (75 µm in diameter) (Fig. 2A). This is probably due to the growing spheroids becoming too large for the microwells and consequently pushing themselves out of the microwells in early stages of spheroid growth, indicating a lower size limit for spheroid size. The co-culture spheroids had a homogeneous diameter with a standard deviation of less than 5% of their diameter, which is comparable to mono-culture spheroids of the same size cultured by *Kang et al*.^18^. It could be observed that the co-culture spheroids inside of the microwells reduced in sizes after three days of culture (Fig. 2D). This observation corresponds to the observations of spheroid formation made by *Lin et al*.^38^. Three stages in spheroid formation were observed, an initial rapid aggregation, a delay period, and a final tight compaction phase. While the first rapid phase is usually completed within the first 12 hours, it can take up to 36 hours for the final phase to complete. In this paper, the first measurement of spheroid size falls within the delay period after the first aggregation phase. The second measurement of spheroid size, however, is after the final aggregation phase which leads to the observed decrease in spheroid size. The uniform-sized multicellular tumor spheroid as a homogeneous model can help to study the effect of anticancer drug-loaded nanoparticles on size or viability of spheroids as previously described^18^. In addition, the homogeneous and uniform-sized 3D tumor spheroids generated in the hydrogel microwells show tight cell-cell junction compared to heterogeneous models as previously described^18^.

**Figure 3:**
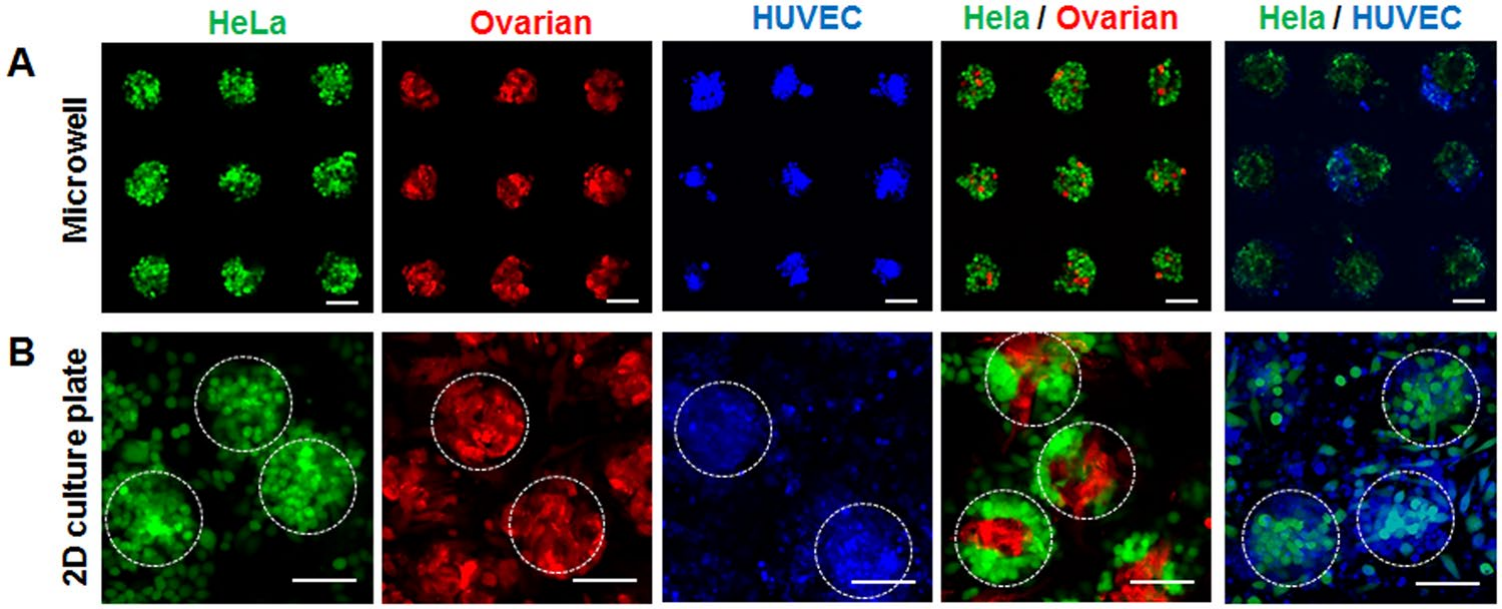
Flourescent micoscopy image of tumor spheroids, consisting of HeLa (green), Ovarian (red), HUVEC (blue), and co-cultures of HeLa/Ovraian and HeLa/HUVEC inside of the 150 µm hydrogel microwells after one day of culutre (**A**) and after replating to a 2D substrate (**B**). Scale bars are 100 µm, white circles indicated individual harvested spheroids.

Here, we decided to harvest the spheroids onto polystyrene confocal microscopy dishes after one day for the final aggregation steps to occur on the substrate that will be used for PTT experiments. Figure 3 shows spheroids in 150 µm microwells before and after harvesting. We observed that the spheroids get distorted in their shape when changing from a 3D hydrogel microwell to a 2D culture substrate. This can be attributed to the insufficient support from the 2D substrate. The confocal microscopy images showed that both HeLa and Ovarian spheroids, as well as the HeLa/Ovarian co-culture spheroids, retained their original sphere shape well after replating. This illustrates that our approach can be used to produce reproducible, uniform sized co-culture spheroids in large quantities, which is challenging with conventional methods. In contrast, HUVEC and HUVEC/HeLa co-culture spheroids became significantly more disturbed in their shape. This can be explained by HUVEC cells, showing a lower tendency to self-aggregate, further HUVEC spheroids are slower to form strong intercellular connections than HeLa and Ovarian cells^39^. Despite these morphological deformations, all spheroids preserve their 3D characteristics as shown by confocal z-stack microscopy (Supplemental Figure 1).

### Synthesis of Nanoparticles

**Figure 4:**
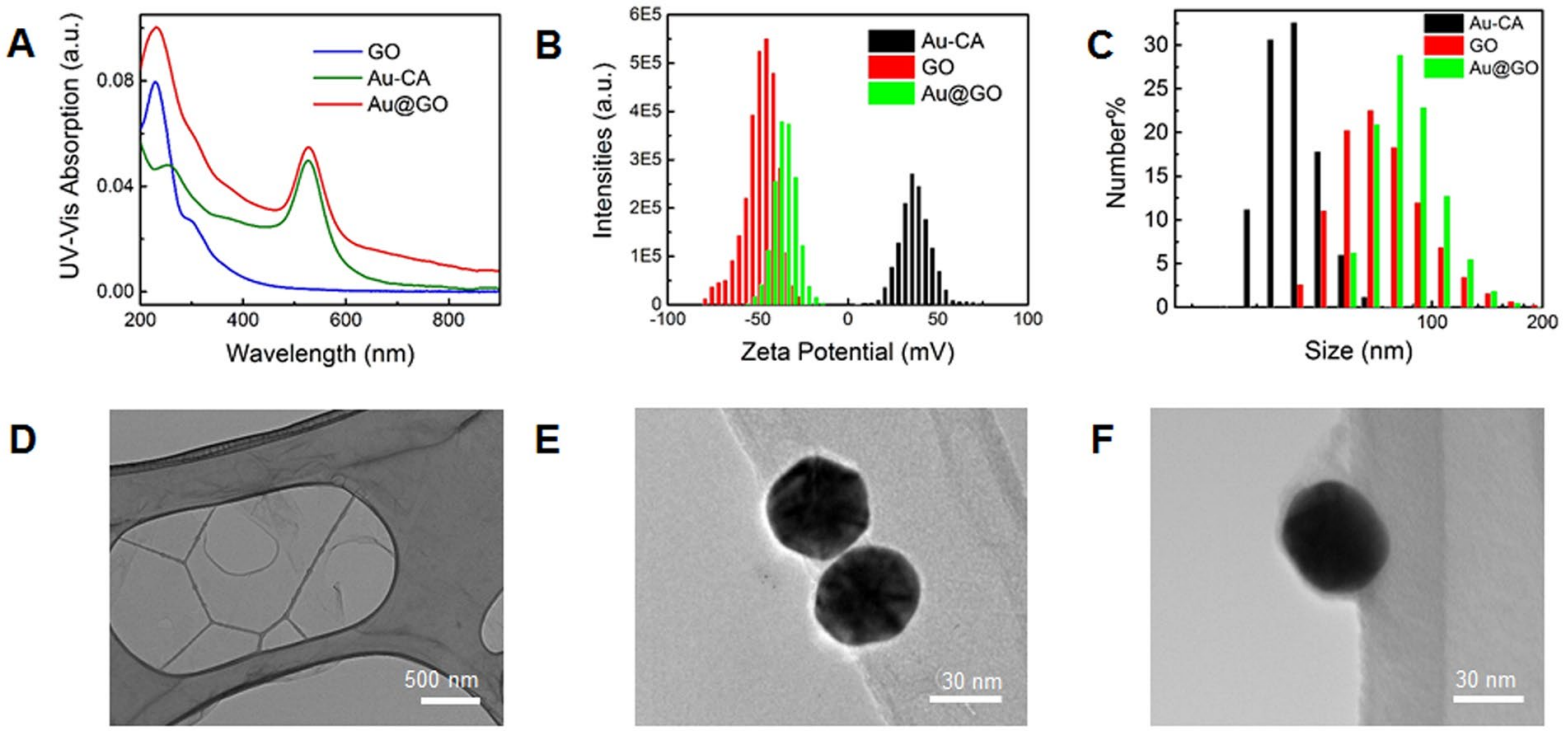
Characterasation of the Au@GO nanomaterial. UV-Vis spectra of GO, Au-CA nanoparticle, and Au@GO nanoparticle (**A**), corresponding Zeta potentials (**B**), and size distributions (**C**). TEM images of GO (**D**), gold nanoparticles (**E**), and Au@GO nanoparticles (**F**).

To investigate the therapeutic potential of hybrid nanomaterials using our generated 3D spheroid co-culture model, we synthesized 40 nm hybrid Au@GO nanoparticles with a core-shell structure. This core-shell nanoparticle was synthesized based on the electrostatic assembly between positively charged gold nanoparticles (+32 mV, cysteamine functionalized) and negatively charged nano-sized GO (110 nm, −49 mV). A 40 nm was selected to achieve a balance between cellular uptake and an efficient photothermal therapy^40^. To obtain the positively charged gold nanoparticles, cysteamine was used simultaneously as a reductant and capping reagent in an aqueous based reaction with HAuCl_4_. Nano-sized GO with high oxidation levels, on the other hand, was synthesized by a modified Hummer’s method. Electrostatic assembly of these two nanoparticles was initiated by the slow addition of positively charged gold nanoparticle solution into a concentrated GO solution. A reversed addition sequence from GO to gold nanoparticles would lead to unstable colloidal aggregations with large sizes, which would be less suitable for delivery-based applications as well as for cancer targeting. The successful formation of our core-shell hybrid structure was characterized by UV-Vis spectroscopy, zeta potential, hydrodynamic sizes, and TEM, respectively (Fig. 4A–C). In the UV-Vis spectrum collected from the Au@GO nanoparticles, it shows two representative absorption peaks from gold nanoparticles at a wavelength of 530 nm (surface plasmon resonance peak) and GO at a wavelength of 225 nm (π-π* transition)^41^. Meanwhile, after encapsulated by the GO to form the Au@GO nanoparticles, the positive zeta potential of cysteamine functionalized Au nanoparticles dramatically changed from a highly positive charge of 32 mV to a negative value of −35 mV, indicating the successful GO coating on its surface. This encapsulation of GO on gold nanoparticles is also supported by an increase of the hydrodynamic size of the Au@GO nanoparticles to 92 nm, as compared to the GO (81 nm) and gold nanoparticles (45 nm). TEM images of Au@GO nanoparticles provide more detailed evidence on the core-shell hybrid structure, where thin-layered GO films enwrap the surface gold nanoparticles with a significantly different shape as compared to TEM images of individual GO or gold nanoparticles (Fig. 4D–F). These characterizations of Au@GO nanoparticles collectively well support the successful synthesis of Au@GO nanoparticles assembled from positively charged gold nanoparticles and negatively charged GO.

**Figure 5:**
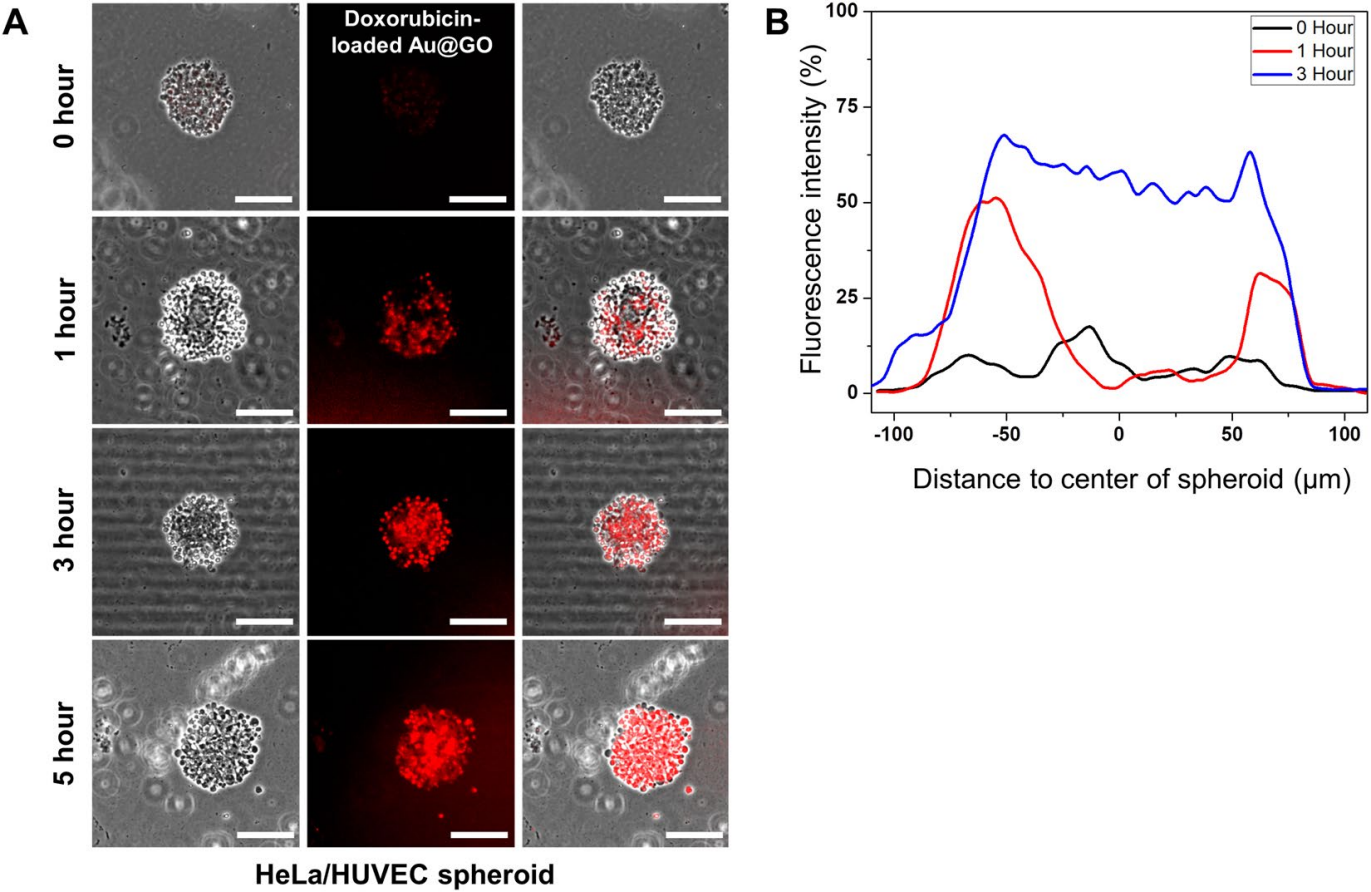
Diffusion analysis of doxorubicin-loaded Au@GO nanoparticles in HeLa/HUVEC spheroids. Diffusion images of doxorubicin-loaded Au@GO nanoparticles in HeLa/HUVEC spheroids for 0, 1, 3, and 5 hours (**A**). Scale bars are 100 μm. Analysis of the fluorescence intensity of doxorubicin-loaded Au@GO nanoparticles in HeLa/HUVEC spheroids (**B**).

Although the spheroid is reported to be an effective tool for the analysis of tumor activity and drug efficacy^42,43^, it has been reported that a the tight morphology of spheroids can hinder diffusion through a lack of extracellular matrix (ECM) compared to *in vivo* tissues^44^. Hence, the development of the ECM within HeLa/HUVEC spheroids was tested by immunostaining of E-cadherin marker (Supplemental Figure S2). E-cadherin is a cell adhesion molecule that is involved in the establishment of cell-cell and cell-ECM interactions, and thus suitable to visualize the formation of ECM within the spheroids^38,45^. It could be shown that the expression of E-cadherin within the 3D HeLa/HUVEC co-culture spheroids is higher compared to 2D cultures. Further we analyzed the diffusion of doxorubicin-loaded Au@GO hybrid nanoparticles within uniform-sized HeLa/HUVEC spheroids (Fig. 5A). As expected, the outer areas of the spheroid showed presence of the nanomaterial after one hour of incubation in doxorubicin-loaded Au@GO growth medium. At the same time the center of spheroids showed only small presence of nanomaterials. This suggest a diffusion gradient from the outside to the center of the spheroids at this early stage of diffusion. After an additional two hours an even distribution of nanomaterials throughout the spheroid can be observed, displaying no gradient anymore (Fig. 5B). This complete saturation of the spheroids remained stable, even after two further hours of incubation. These results indicate that the ECM in the spheroids is sufficiently developed to allow for facile diffusion of nanomaterials. It suggests the feasibility of the 3D, uniform-sized, co-culture spheroids for screening advanced anti-cancer drugs by better mimicking 3D microenvironments in tumors.

### Therapeutic Effects of Photothermal Treatment on Spheroids

**Figure 6:**
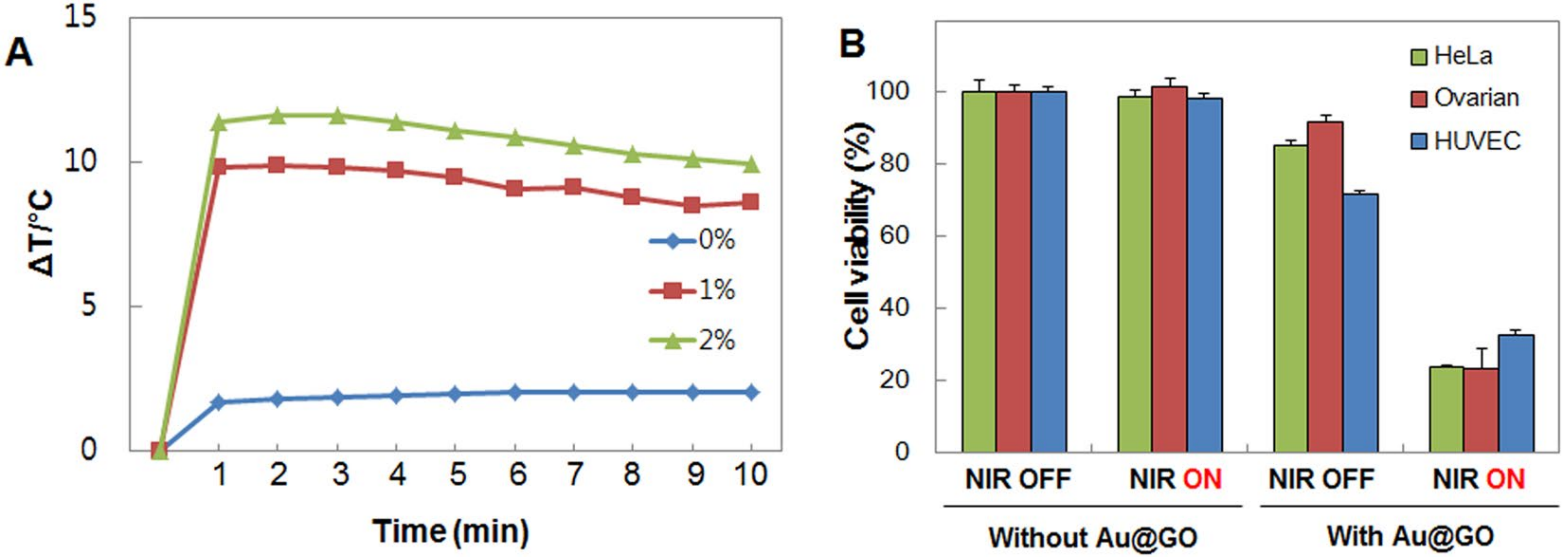
Photothermal effect of the Au@GO nanoparticle. Photothermal heating with different concentrations of Au@GO nanoparticles (**A**). Cell viability of HeLa, Ovarian, and HUVEC cells after NIR treatment with Au@GO nanoparticles and control experiments without the addition of Au@GO nanoparticles (**B**).

A temperature of above 47 °C is desirable to achieve thermal ablation of the cancer cells^46^. First, it was tested which concentration of Au@GO nanoparticles is required to reach this temperature starting. As shown in Fig. 6, after one minute of NIR irradiation, temperature differences of 10 °C (starting from body temperature at 37 °C) were reached, with only 1 vol% of Au@GO nanoparticles. Increasing the concentration to 2 vol% only leads to a marginally higher temperature, which is not relevant for PTT applications. Hence, a concentration of 1 vol% was chosen for the following experiments. Subsequently, PTT experiments with 2D mono-cultures were carried out to test the photothermal effect on the individual cell types (Fig. 6B). Control experiments without the addition of Au@GO nanoparticles showed no negative effects of NIR irradiation on cell viability in 2D cultures. The addition of Au@GO nanoparticles leads to a low dark toxicity before NIR treatment as also observed in toxicity experiments (Supplemental Figure S3). After NIR treatment, the viability of cells reduced drastically to 20% in the case of HeLa and ovarian cells, and 32% in the case of HUVEC cells. These results were confirmed by fluorescent microscopy images of the corresponding spheroids before and after NIR treatment (Supplemental Figure S4). In the fluorescent microscopy images, it was observed that the HUVEC spheroids displayed a higher viability after NIR treatment compared to HeLa, and Ovarian spheroids similar to the observations made in 2D cultures. We believe that the difference in viability could be explained by two different mechanisms. It was previously found that fast dividing cells, such as HeLa cells, have a faster uptake of nanomaterials^47^, which could synergistically increase their sensitivity to our photothermal treatment conditions. Another possible explanation for the lower sensitivity of HUVEC cells could be a higher thermo-sensitivity for fast-dividing cancer cells^48,49^.

**Figure 7:**
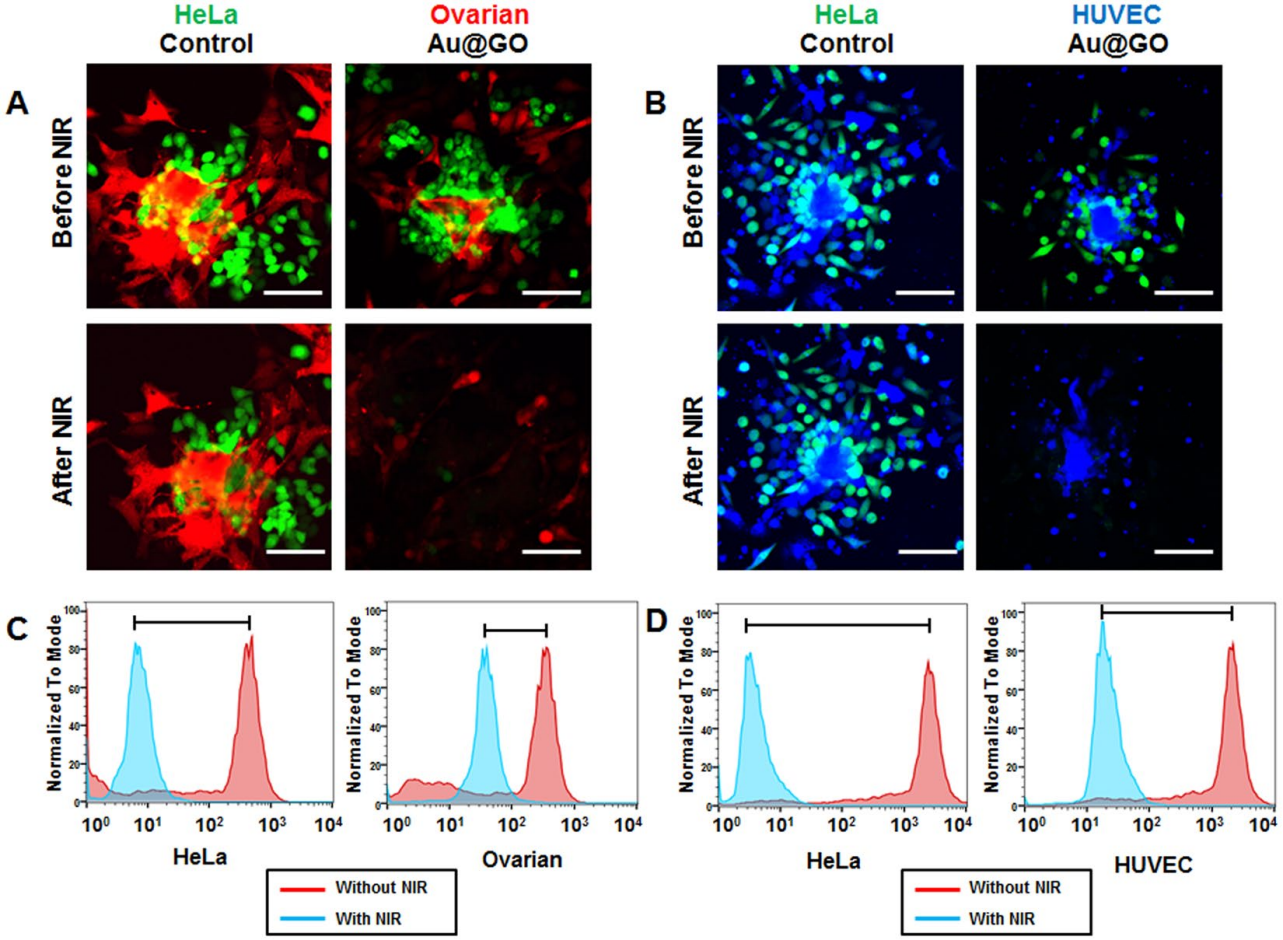
Analysis on PTT-treated co-culture spheroids. Confocal fluorescent microscopy images of HeLa (Green)/Ovarian (Red) co-culture spheroids before and after NIR treatment (**A**). Confocal flurescent microscopy images of HeLa (Green)/HUVEC (blue) co-culture spheroids before and after NIR treatment (**B**). FACS analysis of cell viability of HeLa/Ovarian co-culture spheroids in PTT (**C**). Analysis of cell viability by FACS of HeLa/HUVEC co-culture spheroids in PTT (**D**). All scale bars are 100 µm.

HeLa/HUVEC co-culture spheroids were used as a tumor/endothelium model, and HeLa/Ovarian co-culture spheroids were used as a model for metastasis in carcinomas in experiments testing the photothermal effect of the nanomaterial in 3D tissue models (Fig. 7). As in the experiments using 2D cultures and mono-culture spheroids, NIR irradiation without the addition of Au@GO nanoparticles had no observable effects on the cell viability, as seen from the fluorescent confocal microscopy images (Fig. 7A,C). Conducting PTT experiments with 1 vol% Au@GO nanoparticles and the co-culture spheroids leads to similar results as already observed in 2D cultures and mono-culture spheroids. However, the deviations in the viability of Ovarian and HUVEC cells could be observed. The PTT efficiency on HeLa cells was high for both types of co-culture spheroids killing all HeLa cells, as expected from previous experiments. The viability of Ovarian cells, however, was higher than expected from previous experiments. While in 2D culture experiments the PTT effect of Au@GO nanoparticles on Ovarian cells was similar to the effect on HeLa cells, with the same cell viability after PTT, the viability was higher in 3D co-culture spheroids (Fig. 7A,B). Additionally, we could also observe a higher survival rate of HUVEC cells in HeLa/HUVEC co-culture spheroids (Fig. 7C,D). The higher PTT effect of the Au@GO nanoparticles in the co-culture situation most likely is due to the higher thermo-sensitivity of the fast-dividing HeLa cells^48,49^. A further explanation might be competitive uptake from HeLa cells, reducing the amount of particle samples that can be taken up by HUVEC or Ovarian cells. This effect would be stronger in 3D cultures compared to 2D cultures, as there are more directly neighboring cells competing for uptake.

## Conclusions

In conclusion, addressing critical challenges of 3D spheroids based novel anti-cancer therapeutics screening, we developed a hydrogel microwell array-mediated approach for high throughout producing uniform-sized multicellular 3D tumor spheroids. The co-culture spheroids were harvested from the hydrogel microwell array and can be cultured on 2D substrates while retaining their 3D structures. Furthermore, spheroids generated with our hydrogel microwells were successfully used to test the effectiveness of a newly developed Au@GO nanoparticle as PTT agents for cancer therapy. Through the use of the co-culture spheroids, we demonstrated not only a high therapeutic potential of Au@GO nanoparticles for inducing cancer apoptosis, but also a high selectivity of the nanoparticles towards fast-growing cancer cells in 3D culture. Overall, this method of generating uniform-sized multicellular 3D tumor spheroids could be a powerful tool for *in vitro* drug screening applications.

## Electronic supplementary material

Supplemental Figures
