# Supplementary material for: Generation of uniform-sized multicellular tumor spheroids using hydrogel microwells for advanced drug screening
Source: Sci Rep. 2018 Nov 21;8:17145. doi: 10.1038/s41598-018-35216-7 (PMC6249215; doi:10.1038/s41598-018-35216-7)
Supplement: Supplementary file 1 — Supplemental Figures [file 41598_2018_35216_MOESM1_ESM.docx]

**Supplementary Information**

**Generation of uniform-sized multicellular tumor spheroids using hydrogel microwells for advanced drug screening**

Jong Min Lee^†,1^, Da Yeon Park^†,2^, Letao Yang^†,3^, Eun-Joong Kim^4^, Christian D. Ahrberg^4^,

Ki-Bum Lee^3,*^, Bong Geun Chung^1,*^

^1^ Department of Mechanical Engineering, Sogang University, Seoul, Korea

^2^ Department of Biomedical Engineering, Sogang University, Seoul, Korea

^3^ Department of Chemistry and Chemical Biology, Rutgers, The State University of New Jersey, USA

^4^ Research Center, Sogang University, Seoul, Korea

^†^ These authors contributed equally to this work

**Supplemental Figures**

**
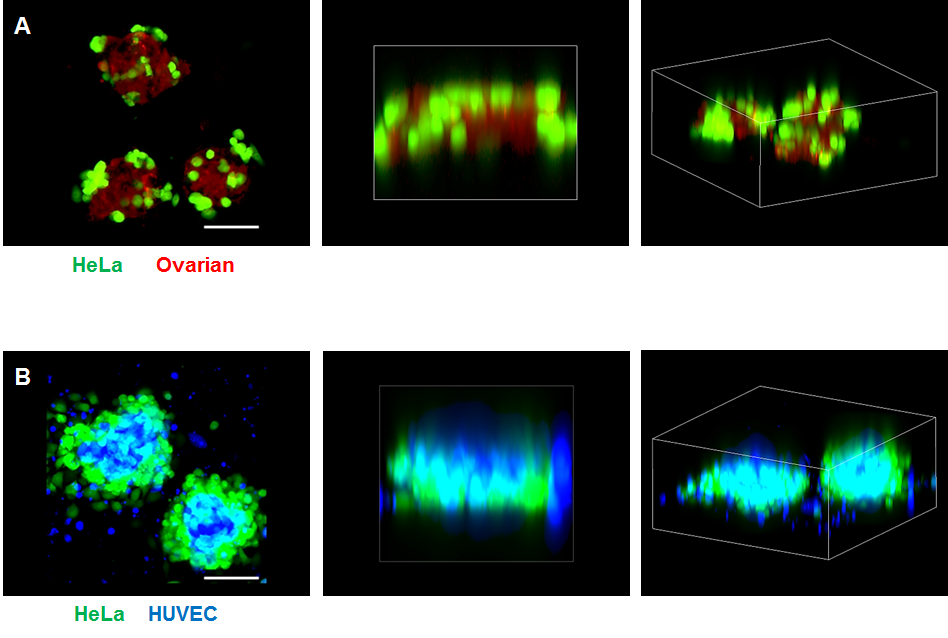
**

**Supplemental Figure S1.** Confocal fluorescent z-stack images of HeLa (green) and Ovarian (red) co-culture spheroids after replating onto the 2D substrate with view from the top (left), the side (middle) and the constructed 3D model of the spheroids (right) (A). Z-stack images of HeLa (green) and HUVEC (blue) co-culture spheroids after replating with a perspective from the top (left), the side (middle) and the 3D model of the spheroids (right) (B). The confocal z-stack images demonstrate that the 3D character of the spheroids is retained after replating onto the 2D substrate. All scale bars are 100µm.


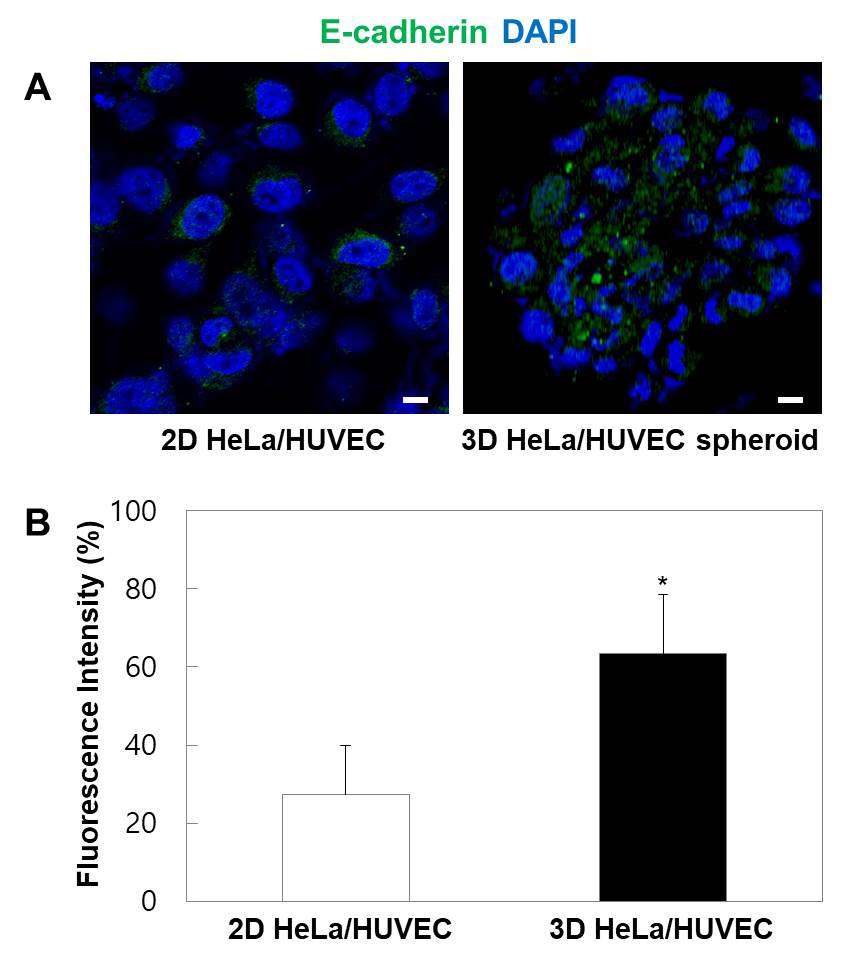


**Supplemental Figure** **S2.** E-cadherin fluorescence images and analysis of 2D and 3D HeLa/HUVEC co-culture spheroids. Confocal fluorescent microscopy images of 2D and 3D HeLa/HUVEC co-culture spheroids (A). Scale bars are 10 µm. Cells are immunostained by E-cadherin (green) and cell nuclei are stained by DAPI (blue). Analysis of E-cadherin expression in 2D and 3D HeLa/HUVEC co-culture spheroids (B). The fluorescence intensity of E-cadherin is normalized by the fluorescence intensity of DAPI in each case.


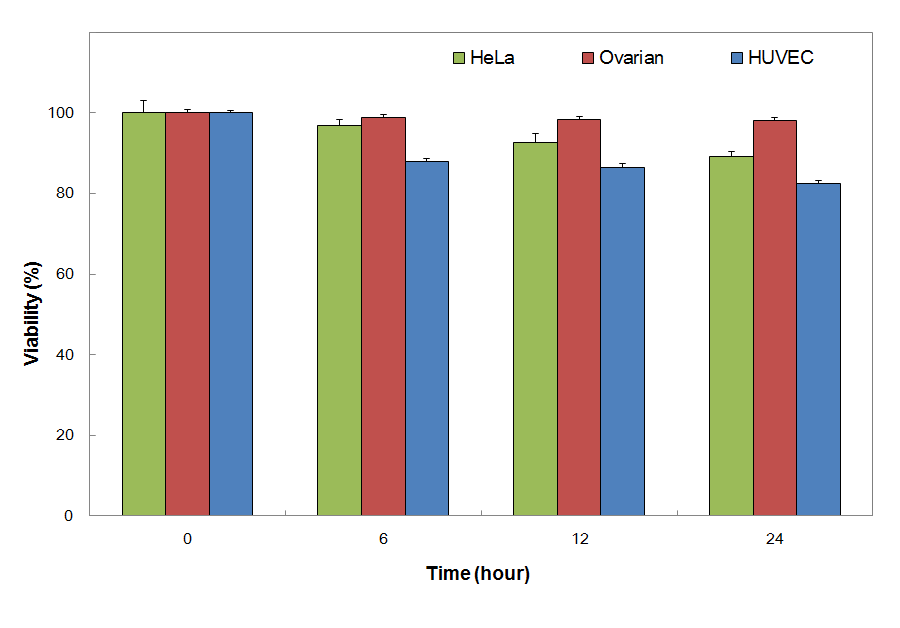


**Supplemental Figure S3.** Dark toxicity of 1 vol% Au@GO nanoparticles on HeLa, Ovarian and HUVEC cells after 0, 6, 12, and 24 hours.


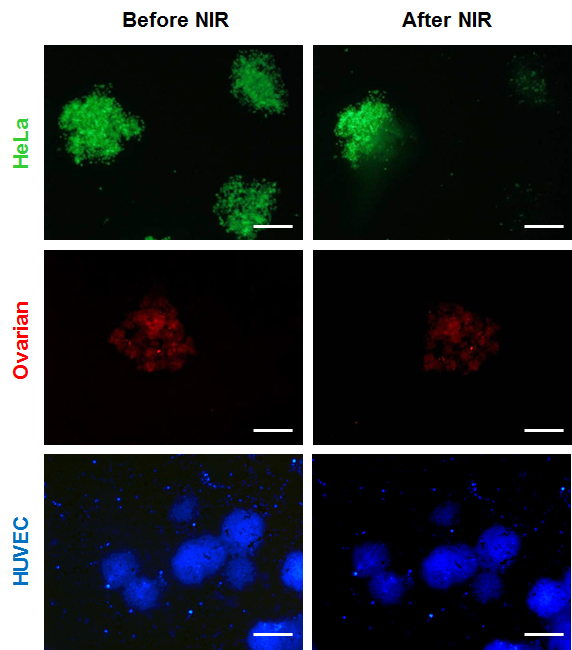


**Supplemental Figure** **S4.** Photothermal effect of 1 vol% Au@GO nanoparticles on HeLa (Green), Ovarian (Red), and HUVEC (Blue) mono-culture spheroids. Confocal fluorescent microscopy images show viability of spheroids before and after NIR irradiation. All scale bars are 100µm.
